# EHMTI-0175. Analysis of migraine among Nepalese population: classification, triggering factors, prophylactic and abortive treatment

**DOI:** 10.1186/1129-2377-15-S1-G28

**Published:** 2014-09-18

**Authors:** R Ojha, R Paudel, DB Shah, A Shrestha, S Koirala, K Adhikari, P Wagle

**Affiliations:** 1 Neurology, National Academy of Medical Sciences Bir Hospital, Kathmandu, Nepal; 2 Neuroscience, Grande International Hospital, Kathmandu, Nepal

## Introduction

Migraine is a chronic neurological disease that is disabling to most of the patients. Although migraine is very common, only a few studies have been reported from Nepal.

## Aims

This study was conducted to understand the characteristics of migraine among Nepalese population.

## Methods

We retrospectively studied migraine patients presented in Neurology outpatient department of Grande International Hospital and Bir Hospital, Kathmandu, Nepal between August 2013 and March 2014.

## Results

A total of 187 migraine patients were included in the study (Grande International Hospital: 138 patients; Bir Hospital: 49 patients). Mean age of the patients was 35.93±13.22 years (range: 8-73 years) with female patients: 147 (78.6%) and male patients: 40 (21.4%). Migraine without aura (73.3%), Migraine with aura (16.0%) and vertebrobasilar migraine (7.5%) are the common migraine types. Thirty-three patients (17.6%) presented as chronic daily headache and 2 patients (1.1%) presented as status migrainosus. Divalproex was the most commonly used as prophylactic treatment (31.0%, followed by propranolol (17.1%) and amitriptyline (17.1%). No prophylactic treatment needed for 37 patients (19.8%), whereas 5 patients (26.7%) needed 2 drugs combination for better control of migraine attacks. Sumatriptan was most commonly used for abortive treatment (89.3%). Sunlight or hot environment was the most common triggering factor (30 patients; 16%).

## Conclusions

Prophylactic medications have a good control over migraine attacks. Avoidance of trigger may further help in the proper management of migraine. Government should further focus on public awareness programs regarding diagnosis and management of migraine in rural areas of Nepal.

No conflict of interest.

